# Ultrasound of the Lacrimal Gland in Healthy Shih Tzu Dogs and with Dry Eye Disease

**DOI:** 10.1155/2024/8516724

**Published:** 2024-09-16

**Authors:** Carla Amorim Neves, Wanessa Patrícia Rodrigues da Silva, Andre Ribeiro Fayad, Beatriz Zanon Ignácio, Aline Maria Vasconcelos Lima, Naida Cristina Borges

**Affiliations:** Escola de Veterinária e Zootecnia Universidade Federal de Goiás, Rodovia Goiânia–Nova Veneza, km 8, Campus Samambaia, Goiânia 74690-900, Goiás, Brazil

## Abstract

Dry eye disease (DED) is a very common disease in dogs, especially that of immune-mediated origin, with Shih Tzu dogs being some of the most affected. In this study, ultrasound changes of the orbital lacrimal gland (LG) were described in healthy Shih Tzu dogs and those diagnosed with different intensities of DED. For training purposes, a macroscopic and ultrasonographic study of the orbits of brachycephalic dog cadavers (*n* = 6) was conducted. Subsequently, thirty-five eyes from 23 male and female dogs aged between two and eight years, distributed into four groups according to the Schirmer tear test (STT-1), including a control group (CG, *n* = 10) (STT-1 ≥15 mm/min), early DED group (DED1, *n* = 8) (STT-1 between 14 and 11 mm/min), moderate DED group (DED2, *n* = 7) (STT-1 between 10 and 6 mm/min), and severe DED group (DED3, *n* = 10) (STT-1 ≤5 mm/min), were used. Ultrasound evaluation of LG showed a higher occurrence of glandular tissue heterogeneity (*p*=0.755) and the presence of hyperechoic areas (*p*=0.027) in dogs with DED3. The variables measuring the major axis were lower in dogs with DED3 (*p*=0.002). The area and perimeter were smaller in dogs with DED3 than in CG and DED1 groups (*p* < 0.001) and the perimeter of the LG of dogs in the DED2 group was smaller than that of CG (*p* < 0.001). The LG major axis (*r* = 0.56), area (*r* = 0.66), and perimeter (*r* = 0.66) decreased as the STT-1 of the different groups decreased. LG ultrasound of Shih Tzu dogs is a feasible technique that allows the evaluation of characteristics of the glandular tissue. The ultrasound could identify changes in texture, echogenicity, and size in LG of Shih Tzu dogs with immune-mediated DED.

## 1. Introduction

Dry eye disease (DED) or keratoconjunctivitis sicca is a progressive and chronic inflammatory ophthalmic disease common in dogs and humans, causing discomfort, ocular itching, inflammation of the ocular surface, and reduced visual acuity, which may culminate in vision loss [1, 2].

Similar to what occurs in humans, DED in dogs often results from dysfunction of the lacrimal glands resulting from immune-mediated dacryoadenitis [1]. DED that occurs as a result of this glandular disease is common in brachycephalic dogs, including Shih Tzu [3–6]. In addition to dacryoadenitis, other diseases can compromise the lacrimal gland in dogs and promote DED, such as hypoplasia, cysts, and neoplasms of the lacrimal glands [1].

The orbital lacrimal gland (LG) is responsible for most of the production of the aqueous portion of the precorneal tear film in the dog [1]. Morphological assessment of LG can be a useful tool to understand glandular structural changes and estimate a prognosis for patients with DED. However, access to canine LG is difficult due to its location in the dorsolateral region of the orbit, deep to the orbital ligament [7].

Difficulty in accessing LG also occurs in humans, but the morphological evaluation of LG has been described through histopathology after incisional and excisional biopsy [8, 9], computed tomography [10], magnetic resonance imaging [11, 12], and ultrasound [13–15]. Ultrasound examination of LG in humans has proven to be a noninvasive, easy-to-perform method and a useful tool for evaluating glandular involvement in patients with DED [13, 15].

LG assessment in dogs using ultrasound has been described in case reports of cysts [16, 17] and neoplasms [18] that compromised the glandular tissue. However, no studies were found in the literature on the ultrasound appearance of LG in dogs with DED or healthy dogs.

The study of the ultrasound characteristics of LG in dogs can provide information on normal glandular morphology for comparative studies in cases of glandular dysfunction. The identification of ultrasound changes of LG in dogs with DED may contribute to the investigation of the cause of the glandular lesion, differentiating acute and chronic inflammatory conditions, fibrosis, neoplastic infiltrations, and cysts, which can contribute to the therapeutic choice and definition of a prognosis. Moreover, LG ultrasound can assist in carrying out invasive procedures, such as obtaining samples for cytology or histopathology.

Considering all aspects, this study aimed to describe ultrasound changes of the orbital lacrimal gland in healthy Shih Tzu dogs diagnosed with different intensities of dry eye disease.

## 2. Materials and Methods

The project was submitted and approved by the Ethics Committee on the Use of Animals (CEUA) at UFG under protocols number 064/18 and 080/19. Those responsible for the animals evaluated in the study authorized the procedures through the Informed Consent Form.

The study was divided into two experimental phases. The first phase consisted of studying six cadavers of healthy brachycephalic dogs, and the second phase consisted of the distribution of 23 Shih Tzu dogs into groups according to the Schirmer tear test 1 (STT-1) values.

### 2.1. Macroscopic and Ultrasound Study of the Orbit of Brachycephalic Dog Cadavers

The ultrasound examination was performed by a single examiner (C. A. N., first author). The ultrasound technique was trained before the experimental period by studying cadavers of brachycephalic dogs (*n* = 6) aiming to standardize the positioning of the transducer and the ultrasound image of LG.

**Figure 1:**
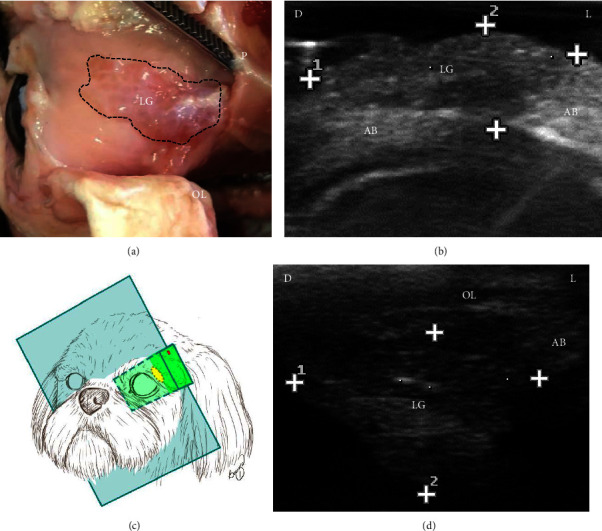
Photograph (a) and ultrasound after dissection (b) of the left orbital lacrimal gland (LG) of a dog cadaver, schematic representation of the transducer positioning (c), and ultrasound of the right lacrimal gland (LG) without dissection of a dog cadaver (d). (a) Demonstration of LG (dotted lines) after section of the orbital ligament (OL) and part of the periorbita (P) and removal of the adjacent adipose body (AB). (b) Hypoechoic LG (between cursors) relative to the surrounding hyperechoic AB. (c) Transducer positioned dorsolateral to the orbit and the dorsal marking (red line). (d) Evidence of homogeneous hypoechoic LG (between cursors), isoechoic AB and part of the hypoechoic OL with hyperechogenic contours. D: dorsal, L: lateral. 18 MHz frequency.

Ultrasound was performed with a Mylab30 Vet device (Esaote®, Genova, Italy) coupled to a linear transducer with a frequency selected at 18 MHz to evaluate surface structures. The left orbit of the cadavers was dissected and the glandular tissue was exposed. Subsequently, the transducer was positioned directly over the glandular tissue to identify the delimitation, echotexture, and echogenicity characteristics of LG on the ultrasound. The ultrasound of the nondissected right orbit was then performed to identify LG and adjacent tissues. The transducer was positioned in accordance with studies in humans [13, 15] to evaluate the nondissected orbit, that is, in the dorsolateral direction to the orbit, with the beams in an oblique direction to the orbital ligament to demonstrate LG (Figure 1).

### 2.2. Animal Selection

Twenty-three dogs of male or female and aged between two and eight years, were selected among the animals treated at the Ophthalmology Service of the School of Veterinary and Animal Science at the Federal University of Goiás (EVZ/UFG) and private clinics in the State of Goiás.

All assessments were carried out at a single time. The animals underwent anamnesis and clinical examination to check the degree of hydration, mucous membrane color, capillary refill time, pulse, cardiac and pulmonary auscultation, heart rate, and respiratory rate. An ophthalmological examination was conducted, and each eye was considered an experimental unit. The eyes were distributed into groups according to STT-1 values. Thus, ten eyes with STT-1 ≥ 15 mm/min were included in the control group (CG–eyes without quantitative DED), eight eyes with STT-1 between 14 and 11 mm/min were included in the DED1 group (eyes with early DED); seven eyes with STT-1 between 10 and 6 mm/min were included in the DED2 group (eyes with moderate DED); and ten eyes with STT-1 ≤5 mm/min were included in the DED3 group (eyes with severe DED). Dogs with other ophthalmological diseases, submitted to topical ocular medications within a period of 30 days before the evaluation, and whose DED was attributed to a nonimmune-mediated cause (neurogenic, medication, iatrogenic, congenital, and infectious) were excluded from the study.

### 2.3. Ultrasound of the Orbital Lacrimal Glands

The dogs underwent LG ultrasound by a single examiner (C. A. N., first author). The examination began one minute after the instillation of a drop of anesthetic eye drops (Anestalcon®, Alcon, São Paulo, Brazil).

The animal was restrained in sternal decubitus, with the face facing the examiner and the head positioned by an assistant to facilitate eye centering and minimize sudden movements. LG ultrasound was performed with the eyelids open and acoustic gel was used for a higher contact between the transducer and the eyelid skin. The dorsolateral orbital region was examined according to the previously studied cutting plane.

The ultrasound characteristics evaluation system described by De Lucia et al. [15] which included a definition of the glandular parenchyma, size, homogeneity, presence of hypoechoic and hyperechoic areas, the appearance of a fibrous gland, and fat deposition, was adapted. The LG size was evaluated by measuring the major axis (D1), minor axis (D2), area (A1), and perimeter (P).

The patients' eyelids were cleaned with sterile 0.9% NaCl saline solution at the end of the ultrasound examination to remove excess acoustic gel. Then, the animals with DED were sent for treatment.

### 2.4. Statistical Analysis

The qualitative variables of the ultrasound examination were presented descriptively, in percentage values, and compared with Fisher's exact test. The significance level for data analysis was 10% (*p* < 0.10). The Shapiro–Wilk normality test was used to verify the distribution of quantitative variables.

STT-1 values and ultrasound measurements were presented as mean and standard deviation and maximum and minimum values. Analysis of variance (ANOVA) and Tukey's test were used for comparison between groups. Statistical differences were considered when the probability value was less than 0.05 (*p* < 0.05) in the ANOVA table.

Pearson correlation was used to establish the relationship between STT-1 values and variables D1, D2, A1, and P (−1 ≤ *r* ≤ 1), considering a 1% significance level (*p* < 0.01). The interpretation of results was carried out in accordance with what was described by Mukaka, [19] in which positive or negative *r* values >0.9 indicate a very high correlation, 0.7–0.9 high correlation, 0.5–0.7 moderate correlation, 0.3–0.5 low correlation, and 0–0.3 negligible correlation. The statistical program R (Version–2016, The R Foundation for Statistical Computing) was used for data analysis.

## 3. Results

Dissection of the left orbit and ultrasound of the six cadavers allowed defining the positioning of the transducer to identify LG, as well as the characterization of the ultrasound aspects of the tissue.

Regarding dogs undergoing clinical ophthalmological evaluation, the STT-1 values observed for each group are shown in Table 1.

Ultrasound evaluation showed that all LG of the studied dogs were located ventral to the orbital ligament and dorsolateral to the ocular bulb.  Table 2 shows the frequency of ultrasound characteristics of LG in different groups of dogs.

**Figure 2:**
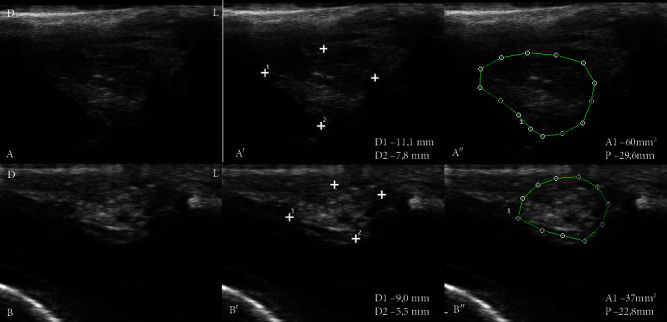
Ultrasound of the orbital lacrimal glands of two male Shih Tzu dogs, belonging to the control group (A, A′, and A″) and with severe dry eye disease (DED3) (B, B′, and B″). Demonstration of homogeneous hyperechoic glandular parenchyma, with poorly defined limits and adjacent hypoechoic structures relative to the orbital lacrimal gland (LG) (A). Higher visibility of LG and increased echogenicity compatible with fibrosis (B). Measurements of major axis (D1), minor axis (D2) (A′, B′), area (A1), and perimeter (P) (A″, B″) of LG (between dots). D: dorsal, L: lateral. 18 MHz frequency.

Ultrasound evaluation of LG from CG showed higher difficulty in defining the parenchyma (30%) compared to LG from dogs with DED, but there was no statistical difference (*p*=0.17) (Figure 2(A)). Glandular tissue heterogeneity was found in all groups, being more frequently evident in dogs with DED3 (60%) and less in CG dogs (10%) (*p*=0.075). The presence of hypoechoic areas was demonstrated in LG with DED3 (20%), DED2 (14.3%), and CG (10%), but there was no significant difference (*p*=0.755). Hyperechogenic areas were present in LG with DED3 (50%), DED2 (42.8%), and DED1 (12.5%), while GC was absent, being more frequent in DED3 (*p*=0.027). The fibrous appearance with diffuse hyperechoic tissue throughout the glandular parenchyma was present in 30% of dogs with DED3 (Figure 2(B)). No ultrasound changes compatible with fat deposition were observed in the glandular tissue.

Table 3 shows the ultrasound measurements of LG of the major axis (D1), minor axis (D2), and the delimitation of the area (A1) and perimeter (P).

The mean values achieved for the variable D1 of LG show a decrease in the size of the measurements (*p*=0.002) in dogs with DED3. The variable D2 of LG did not vary with the disease severity (*p*=0.5359). The variables A1 and P were lower in dogs with DED3 compared to CG and DED1 (*p* < 0.001). Furthermore, the variable P of LG of dogs in the DED2 group was lower than that of CG (*p* < 0.001).

**Figure 3:**
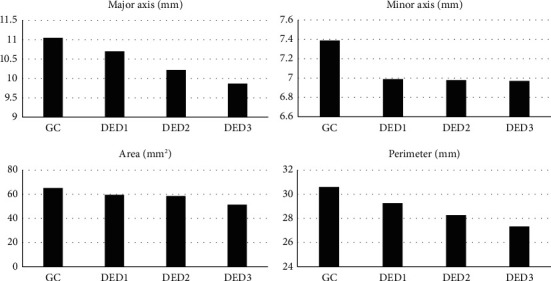
Representation of the variables major axis, minor axis, area, and perimeter of the orbital lacrimal glands of Shih Tzu dogs from the control group (CG, *n* = 10) and those with early (DED1, *n* = 8), moderate (DED2, *n* = 7), and severe (DED3, *n* = 10) dry eye disease (DED).

Figure 3 shows the gradual reduction in the means between the analyzed ultrasound variables. A gradual reduction in the means of D1 and P was observed mainly with a decrease in LG measurements identified in all groups with DED.

The major axis (*r* = 0.56), area (*r* = 0.66), and perimeter (*r* = 0.66) of LG decreased as the STT-1 values of the different groups decreased (*p* < 0.05).

## 4. Discussion

The first phase of this research allowed defining the technique for evaluating LG. Palpation of the orbital ligament indicated to the examiner the positioning area of the transducer to perform the examination. The anatomical characteristic of the dog orbit, whose lateral region is delimited by the orbital ligament and not by bone tissue, allowed obtaining the LG image without the artifact of acoustic shadowing, contrary to what has been observed in humans [13]. In this study, the 18 MHz frequency was sufficient to obtain LG images and allowed the identification of changes in size, echogenicity, and echotexture of the glandular tissue.

The ultrasound images obtained from LG of animals in the DED2 and DED3 groups showed, more frequently, hyperechoic areas, heterogeneous echotexture, and characteristics of fibrosis in the parenchyma. These changes may be related to the severity and chronicity of the dacryoadenitis, which causes DED. Histological studies have evaluated the lacrimal gland in dogs with DED and revealed, in addition to the chronic inflammatory infiltrate, severe glandular degeneration and atrophy, an increase in adipose tissue in the trabeculae, and replacement of the glandular tissue by fibrous connective tissue [20, 21]. De Lucia et al. [15] identified the heterogeneity of the glandular parenchyma and the appearance of the fibrous gland as possible markers of autoimmune dacryoadenitis in humans with Sjögren's syndrome.

The DED3 group also showed a decrease in the major axis, area, and perimeter of LG, which may reflect the greater severity of the disease due to the atrophy described in histopathological studies [20, 21]. Despite the reduction in its size, the identification of LG by ultrasound was easier in DED3 due to the more defined glandular parenchyma when compared to dogs in the DED1 group. It was possibly due to the presence of diffuse fibrous tissue in the parenchyma. In contrast to the findings in the present research, Giovagnorio et al. [13] could not visualize atrophied glands in a study carried out with human beings, possibly due to their very small size or the infiltration of fatty tissue, which made it difficult to differentiate them from the adjacent orbital adipose body.

Access to structural changes in LG requires morphological assessment while the diagnosis of immune-mediated DED in dogs is based on clinical and exclusion criteria [1]. In this sense, ultrasound has shown to have the potential to clarify the severity of glandular damage and to contribute to define the real usefulness of anti-inflammatory/immunomodulatory therapy, as it may not be useful in cases in which there is glandular atrophy.

The failure to perform a histomorphological analysis of LG to clearly define the relationship between ultrasound characteristics and tissue lesions is among the limitations of this study. Furthermore, further studies are required aimed at ultrasound monitoring of LG in the same individual to observe the disease evolution, as well as monitoring patients undergoing therapy for DED.

## 5. Conclusion

LG ultrasound of Shih Tzu dogs is a feasible technique that allows the evaluation of characteristics of the glandular tissue. Ultrasound could identify changes in texture, echogenicity, and size in LG of Shih Tzu dogs with immune-mediated DED.

## Figures and Tables

**Table 1:** Means and standard deviation (SD) and maximum and minimum values of the Schirmer tear test (STT-1) of the eyes of Shih Tzu dogs from the control groups (CG) and those with early (DED1), moderate (DED2), and severe (DED3) dry eye disease (DED).

Groups	Means (mm/min)	STT-1	Maximum (mm/min)	Minimum (mm/min)
		SD		
GC (*n* = 10)	22.60	2.84	25	19
DED1 (*n* = 8)	12.13	1.36	14	11
DED2 (*n* = 7)	8.57	1.51	10	6
DED3 (*n* = 10)	2.40	1.78	5	0

**Table 2:** Ultrasound characteristics of the orbital lacrimal glands of Shih Tzu dogs from the control groups (CG) and those with early (DED1), moderate (DED2), and severe (DED3) dry eye disease (DED).

Ultrasound characteristics	Groups				*p* ^∗^
	GC (*n* = 10)	DED1 (*n* = 8)	DED2 (*n* = 7)	DED3 (*n* = 10)	
Poorly defined glandular parenchyma	3 (30%)	1 (12.5%)	0	0	0.17
Heterogeneous ecotexture	1 (10%)	1 (12.5%)	2 (28.6%)	6 (60%)	0.075
Hypoechoic areas	1 (10%)	0	1 (14.3%)	2 (20%)	0.755
Hyperechoic areas	0	1 (12.5%)	3 (42.8%)	5 (50%)	0.027
Fibrous gland appearance	0	0	0	3 (30%)	1
Fat deposition	0	0	0	0	1

^∗^ *p* < 0.10: comparison of variables in the different groups with Fisher's exact test.

**Table 3:** Means and standard deviation (SD) and maximum and minimum values, in millimeters, of the variables major axis, minor axis, area, and perimeter of the orbital lacrimal glands of Shih Tzu dogs in the control groups (CG, *n* = 10) and those with early (DED1, *n* = 8), moderate (DED2, *n* = 7), and severe (DED3, *n* = 10) dry eye disease (DED).

Variables	Groups	Means ± SD (mm)	*p* ^∗^	Maximum (mm)	Minimum (mm)
Major axis (mm)	GC	11.05 ± 0.64^a^	0.002	12.00	9.90
	DED1	10.70 ± 0.87^ab^		12.20	9.40
	DED2	10.22 ± 0.46^ab^		10.80	9.60
	DED3	9.87 ± 0.50^b^		10.80	9.10

Minor axis (mm)	GC	7.39 ± 0.63	0,5359	8.20	6.40
	DED1	6.99 ± 0.81		8.20	5.70
	DED2	6.98 ± 0.27		7.50	6.70
	DED3	6.97 ± 0.92		7.80	4.90

Area (mm^2^)	GC	65.20 ± 4,64^a^	<0.001	73.00	59.00
	DED1	59.50 ± 6,23^a^		69.00	48.00
	DED2	58.58 ± 5,44^ab^		64.00	49.00
	DED3	51.30 ± 5,38^b^		60.00	44.00

Perimeter (mm)	GC	30.60 ± 1.34^a^	<0.001	33.60	28.90
	DED1	29.27 ± 1.56^ab^		31.10	26.80
	DED2	28.27 ± 1.52^bc^		30.40	25.60
	DED3	27.33 ± 1.14^c^		28.90	25.70

^∗^^,a,b,c^: difference between the means (Tukey's test) of the variables in the different groups (*p* < 0.05).

## Data Availability

All important data from this research are in the article.
